# Applying the Dynamic Dual Pathway Model of Approach Coping to Collective Action Among Advantaged Group Allies and Disadvantaged Group Members

**DOI:** 10.3389/fpsyg.2022.875848

**Published:** 2022-06-06

**Authors:** Helena R. M. Radke, Maja Kutlaca, Julia C. Becker

**Affiliations:** ^1^Department of Psychology, University of Edinburgh, Edinburgh, United Kingdom; ^2^Department of Psychology, Durham University, Durham, United Kingdom; ^3^Department of Psychology, Osnabrück University, Osnabrück, Germany

**Keywords:** collective action, social change, protest, allies, LGBTIQ+, group efficacy, group-based anger

## Abstract

We apply the dynamic dual pathway model of approach coping to understanding the predictors of future collective action among a sample of advantaged group allies and disadvantaged group members who were attending a protest. We propose that problem-focused approach coping (i.e., group efficacy beliefs) would be a stronger predictor of future collective action among disadvantaged compared to advantaged group members, and emotion-focused approach coping (i.e., group-based anger) would be a stronger predictor of future collective action among advantaged compared to disadvantaged group members. Data was collected from LGBTIQ+ and heterosexual people (*N* = 189) protesting as part of the 2019 Christopher Street Day Parade in Cologne, Germany. We found that increased group efficacy predicted intentions to engage in future collective action for the rights of sexual minorities among LGBTIQ+ but not heterosexual participants. Increased group-based anger was a predictor of future collective action intentions regardless of which group the participants belonged to. Our findings extend the dynamic dual pathway model by applying it to a sample of advantaged group allies and disadvantaged group members attending a protest using a multiple perspectives approach.

## Introduction

Advantaged group allies, such as White Americans who participate in the Black Lives Matter movement or heterosexual people who protest for LGBTIQ+ rights, can work together with disadvantaged group members (e.g., Black Americans, sexual minorities) to achieve social change (Czopp and Monteith, 2003; Drury and Kaiser, 2014). Yet it is only recently that the psychological literature has sought to understand allies’ motivations to engage in collective action for disadvantaged groups [see Radke et al. (2020), for an overview]. And few studies use a multiple perspectives approach (Dixon et al., 2012; Di Bernardo et al., 2019; Hässler et al., 2020) which simultaneously considers the experiences of both advantaged and disadvantaged group members (Kutlaca et al., 2020a). Moreover, much of the previous research on this topic has focused on intentions to engage in collective action among members of the general population rather than those who are actively protesting as part of a movement. It is important to study these processes among protesters because doing so acknowledges that activists are a psychologically distinct population [see Zucker (2004), for an example], and suggests that participants will engage in future collective action given that they are already participating in this behavior. In this paper, we address these limitations and extend previous theorizing by applying the dynamic dual pathway model of approach coping with collective disadvantage (Van Zomeren et al., 2012) to understanding the predictors of future collective action among LGBTIQ+ and heterosexual people protesting as part of the 2019 Christopher Street Day Parade in Cologne, Germany.

Collective action is broadly defined as any action, such as protesting or signing petitions, taken to improve the status of a group (Wright et al., 1990; Van Zomeren and Iyer, 2009). While initially this definition was used to describe the actions of disadvantaged group members (Wright et al., 1990), more recently collective action has come to include behaviors taken by people who do not belong to this group (Van Zomeren and Iyer, 2009; Van Zomeren et al., 2011; Saab et al., 2015; Droogendyk et al., 2016; Louis et al., 2019; Radke et al., 2020). In this paper, we refer to advantaged group members who engage in collective action for a disadvantaged group as advantaged group allies (allies for brevity) who are participating in allyship or ally behavior.

Based on a meta-analysis conducted by Van Zomeren et al. (2008), the social identity model of collective action (SIMCA) identified the predictors of collective action. These include identification with the disadvantaged group, perceiving and collectively feeling angry about the injustice that the disadvantaged group experiences (group-based anger), and perceiving that the group can achieve the goals of the collective action [group efficacy beliefs; see also Thomas et al. (2012)]. More recently this model has been expanded to include identification with a politicized group and violated moral beliefs (Simon and Klandermans, 2001; Van Zomeren et al., 2018).

The predictors of SIMCA were later formulated as a theory of collective action in the dynamic dual pathway model of approach coping with collective disadvantage (Van Zomeren et al., 2012). This model proposes that there are two distinct approaches which lead to collective action based on Lazarus (1991) theory of emotion and coping; a problem-focused approach through group efficacy beliefs, and an emotion-focused approach *via* group-based anger. Previous research has found that these two processes are distinct but complementary, and can be activated by support for social action (predicting collective action *via* increased group efficacy beliefs) as well as procedural fairness and perceived support from others [predicting collective action *via* increased group-based anger; Van Zomeren et al. (2004)].

This model has largely been used to describe the processes through which disadvantaged group members come to engage in collective action. Nevertheless, we believe that it is also applicable to understanding the predictors of collective action among both advantaged and disadvantaged group members, albeit to differing degrees. Specifically, we propose that the problem-focused approach (*via* group efficacy beliefs) is a stronger predictor of future collective action intentions among disadvantaged compared to advantaged group members. And the emotion-focused approach (*via* group-based anger) is a stronger predictor of future collective action intentions among advantaged compared to disadvantaged group members. We outline our argument for these predictions below.

With regards to the problem-focused approach, Klandermans (1984) proposed that people conduct a cost-benefit analysis when deciding to engage in collective action, weighing up the subjective value of participating in this behavior against their expectations that the goals of the movement will be achieved [see also Olson (1968) and McCarthy and Zald (1977)]. As such, people should engage in collective action when they believe that this behavior will be effective and that collectively, the goals of the movement can be achieved. This is often referred to as group efficacy beliefs (Van Zomeren et al., 2008).

We propose that group efficacy beliefs are particularly relevant to understanding why disadvantaged group members participate in collective action. Disadvantaged group members stand to benefit from collective action more than allies because this behavior seeks to directly improve the status of their group. Similarly, if a movement does not reach its goal, disadvantaged group members have more to lose (in terms of not improving their status and receiving backlash for challenging the *status quo*) than allies who continue to maintain their higher status as an advantaged group member.

Previous research supports this argument. For example, Hornsey et al. (2006) found that individuals who belong to organized political groups focus on the effectiveness of a rally for building an oppositional movement rather than expressing their values and influencing the public. While the two are not synonymous, disadvantaged group members are more similar to people who are involved in organized political groups (compared to those who are not) because they have an ongoing and pre-established commitment to the cause. As such, we propose that disadvantaged group members are more interested in participating in behaviors that seek to directly improve the status of their group (i.e., building an oppositional movement), rather than more peripheral and personal goals (such as expressing their values), which is consistent with group efficacy beliefs. Importantly other research has also found that lower status group members evaluate whether the social system is unstable (Ellemers et al., 1990; Ellemers, 1993; Saguy and Dovidio, 2013; Scheifele et al., 2021) and change is possible (Cohen-Chen and Van Zomeren, 2018) before participating in collective action, echoing the cost-benefit analysis earlier outlined by Klandermans (1984).

We also expect that the emotion-focused approach will be particularly relevant to understanding intentions to engage in future collective action among advantaged group allies compared to disadvantaged group members. Previous research has shown that a moral conviction—defined as a strong and absolute stance on moralized issues—against inequality predicted intentions to engage in collective action against the discrimination that Muslims experience among non-Muslim participants [Van Zomeren et al., 2011; see also Saab et al. (2015)]. These findings suggest that allies—especially those with a strong moral conviction in favor of the disadvantaged group (such as advantaged group members already protesting)—might be particularly responsive when they perceive that the disadvantaged group is being treated in an unfair and immoral way [see also Radke et al. (2020)]. While distinct, moral convictions and outrage are an emotional response like group-based anger and have shown to be related to this construct in previous research (Van Zomeren et al., 2011).

At the same time, the emotion-focused approach might have less predictive value for future collective action intentions among disadvantaged group members. Activists do experience group-based anger (Van Zomeren et al., 2008) but researchers have argued that they may strategically experience and express this emotion depending on the context they find themselves in Groves (1995), and group-based anger might be experienced as a group norm rather than an emotional state [Thomas et al., 2009; see also Van Zomeren (2015)].

We would add here that while group-based anger might be an appropriate and motivating response to initially recognizing an injustice, it is not sustainable in the long-term and can lead to burnout (Gorski and Chen, 2015). As such activists might learn to strategically regulate their experience and expression of group-based anger to ensure the longevity of their participation in the movement. While activists can be both advantaged and disadvantaged group members, they are more likely to be disadvantaged group member (or at least psychologically similar to this group) because the issue they take action for directly affects their lives. Taken together these findings suggest that the emotion-focused approach (*via* group-based anger) would be a stronger predictor of future collective action intentions among both advantaged group allies compared to disadvantaged group members.

The aim of the current study was to apply the dynamic dual pathway model of coping with collective disadvantage (Van Zomeren et al., 2012) to advantaged group allies and disadvantaged group members who were participating in a protest. We propose that the problem-focused approach for disadvantaged group members, and the emotion-focused approach for advantaged group members, would be particularly relevant to understanding intentions to engage in future collective action. We therefore hypothesized that group efficacy beliefs would be a stronger predictor of future collective action intentions among disadvantaged group members compared to allies, and group-based anger would be a stronger predictor of future collective action intentions among allies compared to disadvantaged group members. To test this hypothesis, we collected data from allies (heterosexual people) and disadvantaged group members (members of the LGBTIQ+ community) who protested as part of the 2019 Christopher Street Day Parade in Cologne, Germany.

## Materials and Methods

### Participants

The participants of this study were 234 people who were protesting as part of the 2019 Christopher Street Day Parade in Cologne, Germany. Twenty-nine participants were deleted from the dataset because they could not be categorized as an ally or disadvantaged group member, and 16 participants were deleted because they were not 18 years or older. The final sample size consisted of 189 participants. Power analysis using G*Power (Faul et al., 2007) indicated that a total sample of 199 participants would be needed to detect a small to medium effect (*f* = 0.20, *r* = 0.20) with 80% power and an alpha of 0.05^1, 2^.

The participants had an age range of 18–67 years old, with a mean age of 27.54 years (*SD* = 10.38; 1 participant did not report their age). The sample was comprised of 108 participants (57%) who identified as female, 75 participants (40%) who identified as male, and five participants (3%) who did not identify with either group (one participant did not report their sex). One hundred and twenty-five participants (66%) identified as being a member of the LGBTIQ+ community and 64 participants (34%) identified as heterosexual. One hundred and sixty-six participants (88%) had German citizenship, and 19 participants (10%) did not (four participants did not report their nationality; 2%). The majority of participants had completed high school (*N* = 117; 62%), followed by 62 participants (33%) who completed a university degree, and eight participants (4%) who did not complete high school (two participants did not indicate their highest level of education; 1%).

### Procedure

The data was collected at the 2019 Christopher Street Day Parade in Cologne, Germany. Participants were approached by a team of research assistants working in pairs and asked to complete a short survey about their reasons for attending the protest. The data was collected using pen and paper questionnaires and by giving participants a link to the same study online that they could complete on their phones or a tablet provided by the research assistants. Participants were asked to read the information and informed consent sheet, complete the survey, and then read the debriefing sheet before being rewarded with rainbow stickers and flags as well as the chance to go into a draw to win one of many gift cards. Participants had the option of completing the questionnaire in German or English.

A protocol was developed to standardize the data collection based on previous research conducted by the authors (Ferris et al., 2019; Kutlaca et al., 2020b) and other researchers (Van Leeuwen et al., 2015; Walgrave et al., 2016). Research assistants were instructed to wear t-shirts or stickers on their coats that identified them as being from the university, and to carry their university ID card with them. They were to introduce themselves as a student from the university that was asking protesters to complete a survey about their reasons for attending the march. They were instructed to not give their opinion, give the participants some space when completing the survey, and encourage the participants to complete the questionnaire separately.

The research assistants were instructed to approach the protest from all sides, as well as from the front and back, but to not find themselves in a situation where it would be difficult to exit the crowd quickly. If they were to feel uncomfortable they were told to step away from the crowd, and had the phone numbers of the other research assistants (and the authors) if they got lost or had any difficulties. They were instructed to watch the police as an indicator of what might happen next at the protest. The study was approved by the University of Osnabrück research ethics committee.

### Measures

The following variables were measured on a 1 (*strongly disagree*) to 7 (*strongly agree*) likert-type scale unless otherwise specified.

#### Group Efficacy Beliefs

Group efficacy beliefs was measured using four items (e.g., “I believe that demonstrators, as a group/together/through joint actions, can achieve greater rights for LGBTIQ+ people”; “I believe that demonstrators can reach their common goal of achieving greater rights for the LGBTIQ+ community”; α = 0.96).

#### Group-Based Anger

Group-based anger was measured using three items (e.g., **“**I feel angry/outraged/furious about how LGBTIQ+ people are treated in Germany”; α = 0.92).

#### Future Collective Action Intentions

Future collective action intentions were measured using eight items (e.g., “In the future I would be willing to participate in the following actions to achieve greater rights for the LGBTIQ+ community… attend public talks, discussion meetings, rallies and demonstrations, distribute flyers, sign petitions, strikes, boycott companies, donate money”; α = 0.87) on a 1 (*very unlikely*) to 7 (*very likely*) likert-type scale.

#### Previous Experience Engaging in Collective Action for LGBTIQ+ Rights

Previous experience engaging in collective action for LGBTIQ+ rights was measured using one item (“I regularly attend demonstrations for LGBTIQ+ rights”).

## Results

Means, standard deviations, and correlations between the variables can be seen in Table 1.

**TABLE 1:** Means, standard deviations, and correlations between the variables.

	LGBTIQ+ mean (SD)	Heterosexual mean (SD)	1	2	3	4	5	6
1. Age	26.56 (9.25)	29.44 (12.13)	–	–0.07	−0.29*	−0.28*	–0.07	–0.22
2. Education	3.90 (1.07)	4.02 (0.97)	0.26**	–	0.13	0.16	–0.16	0.05
3. CA experience	4.80 (1.89)	3.48 (1.92)	–0.06	–0.01	–	0.26*	0.07	0.39**
4. Group efficacy	6.16 (1.04)	5.99 (1.20)	–0.01	–0.03	0.34***	–	0.10	0.05
5. Group-based anger	4.33 (1.72)	4.51 (1.74)	–0.08	−0.26**	0.04	0.09	–	0.37**
6. Future CA	4.77 (1.31)	4.12 (1.45)	–0.04	–0.06	0.49***	0.37***	0.20*	–

*p < 0.05, **p < 0.01, ***p < 0.001. Education: Kein Abschuluss/No Education Certificate = 1. Hauptschulabschluss/GCSE General School = 2. Realschulabschluss/GCSE Vocational Training = 3. (Fach-) Abitur/A-Level = 4. Hochschulabschluss/University Degree = 5. Data from LGBTIQ+ participants reported below the diagonal and heterosexual participants above the diagonal. CA = collective action.

We used Mplus version 8.6 to run a moderation model with two predictors (group-based anger and group efficacy), group membership as the dichotomous moderator (advantaged group allies = 0; disadvantaged group members = 1), and future collective action intentions as the outcome (Stride et al., 2015). We opted for maximum likelihood estimation with robust standard errors (MLR). Regression analyses were conducted, the variables were mean-centered, and the analysis was conducted on the observed variables so no model fit indices are reported (see Table 2).

**TABLE 2:** Regression model for group-based anger and group efficacy beliefs predicting future collective action intentions moderated by group membership.

	*B*	*B*_*SE*_	95% CI
**Without control variables**			
Main effect of group-based anger	0.30***	0.09	0.15, 0.46
Main effect of group efficacy	0.02	0.14	−0.22, 0.25
Main effect of group membership	0.67**	0.20	0.34, 1.00
Group-based anger × group membership	–0.18	0.12	−0.37, 0.02
Group efficacy × group membership	0.43*	0.20	0.11, 0.76
Simple slopes for LGBTIQ+ participants	0.45***	0.14	0.22, 0.68
Simple slopes for heterosexual participants	0.01	0.14	−0.22, 0.25
**With control variables**			
Main effect of group-based anger	0.27**	0.08	0.14, 0.41
Main effect of group efficacy	–0.13	0.12	−0.34, 0.07
Main effect of group membership	0.30	0.19	−0.01, 0.62
Group-based anger × group membership	–0.10	0.10	−0.26, 0.07
Group efficacy × group membership	0.37*	0.16	0.11, 0.64
Simple slopes for LGBTIQ+ participants	0.24*	0.12	0.04, 0.43
Simple slopes for heterosexual participants	–0.13	0.12	−0.34, 0.07

*p < 0.05, **p < 0.01, ***p < 0.001. LGBTIQ+ participants = 1. Heterosexual participants = 0. Control variables included age, sex, nationality, level of education, and past collective action. Simple slopes only reported for significant interactions.

**FIGURE 1:**
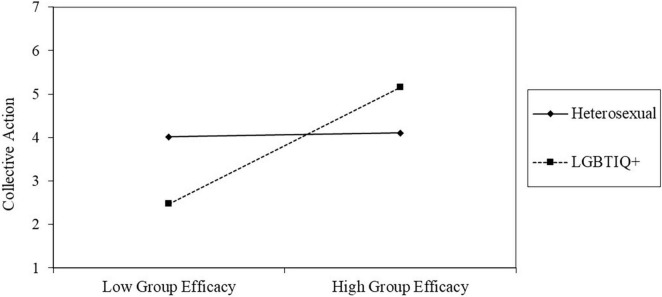
Group efficacy beliefs moderated by group membership on future collective action intentions.

We did find a main effect of group membership (*B* = 0.67, *B*_*SE*_ = 0.20, *p* = 0.001). Moreover, no main effect of group efficacy beliefs on future collective action intentions emerged (*B* = 0.02, *B*_*SE*_ = 0.14, *p* = 0.919). But we did find that group membership moderated this relationship (*B* = 0.43, *B*_*SE*_ = 0.20, *p* = 0.030). In line with our hypothesis, simple slopes analyses revealed that group efficacy beliefs predicted future collective action intentions among disadvantaged group members (*B* = 0.45, *B*_*SE*_ = 0.14, *p* = 0.001) but not allies (*B* = 0.02, *B*_*SE*_ = 0.14, *p* = 0.919; see Figure 1).

We also found a significant main effect of group-based anger (*B* = 0.30, *B*_*SE*_ = 0.09, *p* = 0.001), such that increased group-based anger among both allies and disadvantaged group members predicted intentions to engage in future collective action. But we did not find an interaction between group-based anger and group membership on future collective action intentions (*B* = −0.18, *B*_*SE*_ = 0.12, *p* = 0.133). The overall model was significant (*R*^2^ = 0.19, *p* = 0.002).

It is possible that our results are affected by participants’ previous experience with collective action and other demographic variables (i.e., participant age, sex, nationality, and level of education). We therefore ran the analysis again controlling for these variables. Only previous experience engaging in collective action was significant (*B* = 0.29, *B*_*SE*_ = 0.06, *p* < 0.001) but not the other control variables (*ps* ≥ 0.286).

We did not find a main effect of group membership (*B* = 0.30, *B*_*SE*_ = 0.19, *p* = 0.116). No main effect of group efficacy beliefs on future collective action intentions emerged (*B* = −0.13, *B*_*SE*_ = 0.12, *p* = 0.277) but again we did find that group membership moderated this relationship (*B* = 0.37, *B*_*SE*_ = 0.16, *p* = 0.022). In line with our hypothesis simple slopes analyses revealed that group efficacy beliefs predicted future collective action intentions among disadvantaged group members (*B* = 0.24, *B*_*SE*_ = 0.12, *p* = 0.045) but not allies (*B* = −0.13, *B*_*SE*_ = 0.12, *p* = 0.277).

We also found a significant main effect of group-based anger (*B* = 0.27, *B*_*SE*_ = 0.08, *p* = 0.001), such that increased group-based anger among both allies and disadvantaged group members predicted intentions to engage in future collective action. We did not find that this relationship was moderated by group membership (*B* = −0.10, *B*_*SE*_ = 0.10, *p* = 0.344). The overall model was significant (*R*^2^ = 0.38, *p* < 0.001).

## Discussion

In this paper we applied the dynamic dual pathway model of approach coping with collective disadvantage (Van Zomeren et al., 2012) to understanding the predictors of future collective action among a sample of advantaged group allies and disadvantaged group members who took part in a protest. Specifically, we proposed that the problem-focused approach would be particularly relevant to disadvantaged group members, but the emotion-focused approach would be especially important to advantaged group members. We collected data from heterosexual and LGBTIQ+ people protesting as part of the 2019 Christopher Street Day Parade in Cologne, Germany to test our hypotheses. We found that group efficacy beliefs were a stronger predictor of future collective action intentions among disadvantaged group members compared to allies. However, group-based anger predicted future collective action intentions among both heterosexual and LGBTIQ+ people. This pattern of results held when including the additional control variables.

The finding that group efficacy predicts future collective action intentions among disadvantaged group members but not allies is consistent with our argument that the problem-focused approach would be more relevant to disadvantaged group members. We drew from previous research to make this argument (Hornsey et al., 2006) which found that individuals who belonged to an organized political group (who we argue are more similar to disadvantaged group members because they have an ongoing and pre-existing commitment to the cause) focused on the effectiveness of the rally for building an oppositional movement compared to people who did not belong to an organized political group. This is in line with other research which has found that disadvantaged group members weigh up (Klandermans, 1984) whether change is likely (Ellemers et al., 1990; Ellemers, 1993; Saguy and Dovidio, 2013; Scheifele et al., 2021) and possible (Cohen-Chen and Van Zomeren, 2018) before participating in collective action. Unlike advantaged group members—who will continue to benefit from their higher status if the movement is not successful—disadvantaged group members have a lot to gain (in terms of improved status) but also a lot more to lose (because of the potential for backlash for challenging the *status quo*) depending on the success of the collective action.

Contrary to our predictions, we did not find that group-based anger was a stronger predictor of future collective action intentions among advantaged compared to disadvantaged group members. While we drew on literature about moral convictions (Van Zomeren et al., 2011) and moral outrage (Saab et al., 2015) to build this argument we only measured group-based anger in our study. We chose to do this because group-based anger is included in the dual process model and is more applicable to understanding the experiences of both advantaged and disadvantaged group members. It is possible that we may have found differences between these two groups if we included moral outrage instead of anger in the study, and therefore encourage future research to follow-up this prospect. Likewise, it is also possible that group-based anger is a predictor of future collective action intentions for both groups albeit for different reasons. As discussed, group-based anger might predict future collective action because this is an appropriate initial response to recognizing an injustice among advantaged group members. And disadvantaged group members might express group-based anger as a group norm (Thomas et al., 2009), and strategically to protect against activist burnout (Gorski and Chen, 2015). Future research could also consider other emotional predictors for allies’ participation in collective action such group-based sympathy (Harth et al., 2008) and guilt [Iyer et al., 2003; see also Thomas et al. (2009) and Radke et al. (2020), for further discussion].

The strengths of our study include that we used a multiple perspectives approach (Kutlaca et al., 2020a) to apply the dynamic dual pathway model (Van Zomeren et al., 2012) to a sample of protesters. By using a multiple perspectives approach, new possibilities for understanding the causes and consequences of allyship in an ecologically valid way can be achieved. And while we only measured intentions to engage in future behavior, we did measure this in a sample of participants who were actively participating in a protest. This helps us to further understand the behavior of those who actually attend rallies, and be more confident that these future collective action intentions will be realized.

While recruiting participants from an actual protest event was a strength of the study, it also meant that the study contained a relatively small and unbalanced sample. This is a common constraint associated with data collection during a real-world event where it is limited to a given day and time. We took steps to mitigate this in the data analysis by opting for maximum likelihood estimation with robust standard errors (MLR) as it is a robust to violations of normality and non-independence (Muthén and Asparouhov, 2002). And despite having a small and unbalanced sample we still found partial support for our hypotheses.

Future research could consider recruiting participants who take part in other protests to show that our findings can be replicated and are generalizable. Although our results are promising we are aware that we cannot fully determine whether our findings hold for all disadvantaged group members and advantaged group allies until further data is collected. Nevertheless, we believe that it is still important to publish these results as a step forward toward answering this research question. Our findings provide preliminary support for the usefulness of applying the dual process model to understanding the predictors of collective action taken by a politicized sample of disadvantaged group members and advantaged group allies using a multiple perspectives approach.

## Data Availability Statement

The datasets presented in this study can be found in online repositories. The names of the repository/repositories and accession number(s) can be found in the article/Supplementary Material.

## Ethics Statement

The studies involving human participants were reviewed and approved by University of Osnabrüeck Ethics Committee. The patients/participants provided their written informed consent to participate in this study.

## Author Contributions

HR and MK organized the data collection and performed the statistical analysis. HR wrote the first draft of the manuscript. All authors contributed to the conception and design of the study and manuscript revision and read and approved the submitted version.

## Conflict of Interest

The authors declare that the research was conducted in the absence of any commercial or financial relationships that could be construed as a potential conflict of interest.

## Publisher’s Note

All claims expressed in this article are solely those of the authors and do not necessarily represent those of their affiliated organizations, or those of the publisher, the editors and the reviewers. Any product that may be evaluated in this article, or claim that may be made by its manufacturer, is not guaranteed or endorsed by the publisher.

## Footnotes

1 Data was collected from other protests (Women’s Day March in Münster; Stand Up Against Racism Protest in Berlin) but the sample size was too small to analyze on its own.

2 The study was not preregistered but outlined in a funded grant application (DFG BE 4648/4-2) awarded to the third author.
